# Helical ensembles outperform ideal helices in molecular replacement

**DOI:** 10.1107/S205979832001133X

**Published:** 2020-09-16

**Authors:** Filomeno Sánchez Rodríguez, Adam J. Simpkin, Owen R. Davies, Ronan M. Keegan, Daniel J. Rigden

**Affiliations:** a Institute of Structural, Molecular and Integrative Biology, University of Liverpool, Liverpool L69 7ZB, United Kingdom; b Life Science, Diamond Light Source, Harwell Science and Innovation Campus, Didcot OX11 0DE, United Kingdom; c Institute for Cell and Molecular Biosciences, Newcastle University, Framlington Place, Newcastle upon Tyne NE2 4HH, United Kingdom; d UKRI–STFC, Rutherford Appleton Laboratory, Research Complex at Harwell, Didcot OX11 0FA, United Kingdom

**Keywords:** molecular replacement, *AMPLE*, helical ensembles

## Abstract

The conventional approach in molecular replacement is the use of a related structure as a search model. However, this is not always possible as the availability of such structures can be scarce for poorly characterized families of proteins. In these cases, alternative approaches can be explored, such as the use of small ideal fragments that share high, albeit local, structural similarity with the unknown protein. Earlier versions of *AMPLE* enabled the trialling of a library of ideal helices, which worked well for largely helical proteins at suitable resolutions. Here, the performance of libraries of helical ensembles created by clustering helical segments is explored. The impacts of different *B*-factor treatments and different degrees of structural heterogeneity are explored. A 30% increase in the number of solutions obtained by *AMPLE* was observed when using this new set of ensembles compared with the performance with ideal helices. The boost in performance was notable across three different fold classes: transmembrane, globular and coiled-coil structures. Furthermore, the increased effectiveness of these ensembles was coupled to a reduction in the time required by *AMPLE* to reach a solution. *AMPLE* users can now take full advantage of this new library of search models by activating the ‘helical ensembles’ mode.

## Abstract 2

Helical ensembles solve more structures by MR with *AMPLE* than do ideal helices and at no greater CPU cost.

## 1. Introduction   

X-ray crystallography is the most prevalent technique for protein structure determination (Berman *et al.*, 2002 ▸), but the phase problem remains one of its most challenging aspects. Molecular replacement (MR) is often the method of choice to obtain the missing phase information, primarily because of its speed and high potential for automation (Evans & McCoy, 2008 ▸). This approach relies on replacing the missing experimental phases of the unknown structure with the calculated phases of a similar solved structure (Rossmann, 1990 ▸) positioned appropriately in the unit cell. Thus, the more structurally similar the search model is to the unknown structure, the more probable it is that MR will have a positive outcome. In nontrivial MR cases, the availability and detection of suitable search models can be a key limitation and alternative routes need to be explored. One such route is the use of small fragments such as α-helices. This was first proposed as an efficient phasing technique to solve cases in which high-resolution scattering data were available (Yao, 2002 ▸). Since most proteins contain α-helices or β-strands as secondary-structure elements, such standardized fragments are often a valid approximation to elements of the unknown structure. Nevertheless, the use of these search models has the intrinsic difficulty of low signal-to-noise ratio as they only represent a small fraction of the overall structure. Such complications were addressed by the development of *ARCIMBOLDO* (Rodríguez *et al.*, 2009 ▸), which combined the use of *Phaser* (McCoy *et al.*, 2007 ▸) for the accurate placement of several small ideal fragments with the use of *SHELXE* (Thorn & Sheldrick, 2013 ▸) for electron-density map modification and chain autotracing. The use of this sophisticated approach enabled the solution of increasingly challenging structures with lower resolutions (up to 3 Å) and larger numbers of residues in the asymmetric unit. Additionally, this approach proved to be successful in the solution of a variety of folds, including coiled coils (Caballero *et al.*, 2018 ▸), which are notoriously difficult to solve through MR as they often suffer from different crystallographic data pathologies such as anisotropic diffraction and apparent translational noncrystallographic symmetry (Thomas *et al.*, 2020 ▸). Targets containing as many as 600 residues have been solved in this way (Caballero *et al.*, 2018 ▸). Further developments in fragment-based MR came with *FRAGON* (Jenkins, 2018 ▸). This tool achieves high success rates among high-resolution structures, while having a relatively low consumption of computational resources by taking advantage of the ability of *Phaser* to place small search fragments and of *ACORN*’s sophisticated scoring algorithm for density modification (Yao *et al.*, 2006 ▸).

*AMPLE* first made use of such standard fragments as a baseline to assess the performance of *ab initio* modelling decoys as search models for the solution of the aforementioned challenging coiled-coil structures (Thomas *et al.*, 2015 ▸). This small library of eight ideal helices, with lengths ranging from five to 40 residues, proved to be surprisingly effective as more than half of the structures under study could be solved, thus revealing a fast and simple MR approach. Later studies suggested that the efficacy of *AMPLE*’s ideal helices extends to different fold types, such as α-helical transmembrane proteins (Thomas *et al.*, 2017 ▸). However, despite the relative success observed when using these search models, limitations in the use of these helices as search models could be observed, as revealed by the scarce presence of solved structures with more than 300 residues in the asymmetric unit and diffraction data with a resolution of 2 Å or worse. Nevertheless, such limitations are to be expected, as idealized fragments cannot capture in full the details of biologically active protein folds, which are a result of a combination of biochemical interactions that will cause a series of distortions in the local fold of the structure (Koga *et al.*, 2012 ▸). However, several tools have been developed in the field of fragment-based MR aimed at this and other limitations intrinsic to fragment search models, such as *ARCIMBOLDO_BORGES* (Sammito *et al.*, 2013 ▸), which makes use of multiple fragments to expand the size of the search model into recurring tertiary-structure motifs, *ALEPH* (Medina *et al.*, 2020 ▸), which mines the structures available in databases to create libraries of fragments that can be used as search models, and *ALIXE* (Millán *et al.*, 2020 ▸), which combines phase information from partial solutions originating from these correctly placed fragment search models.

Here, we explore new ways to increase the efficacy of helical fragments as search models in *AMPLE* by making use of ensemble search models. Ensembles of multiple search models created through structural alignment have a long history of outperforming their individual component structures (Bibby *et al.*, 2012 ▸; Chen *et al.*, 2000 ▸; Keegan *et al.*, 2018 ▸; Leahy *et al.*, 1992 ▸; Pieper *et al.*, 1998 ▸; Rigden *et al.*, 2002 ▸; Simpkin *et al.*, 2020 ▸). With ensemble search models, the structural variability of the members in the ensemble can be used to statistically weight sets of structure factors (Read, 2001 ▸). We observe that a new set of 64 helical ensembles created by mining the currently available structures in the PDB (Berman *et al.*, 2002 ▸) and superimposing detected helices significantly outperforms the original set of single model ideal helices as search models. Additionally, we compare MR success rates for ensembles with different levels of structural heterogeneity and subjected to different *B*-factor treatments. Compared with the original set of ideal helices, we registered a 30% increase in the total number of solutions when using the new library of helical ensembles. Improvements were seen in all three groups: transmembrane, globular and coiled-coil folds. This increase in the number of MR successes is coupled with a decrease in the time elapsed before reaching the first solution for a given structure when using a minimal subset of 12 ensembles.

## 2. Materials and methods   

### 2.1. Data-set selection   

The test set of globular and transmembrane structures was selected by first retrieving a list of all of the X-ray PDB entries with a resolution between 2.0 and 3.0 Å, from which chain lengths of between 100 and 700 residues were selected. Transmembrane protein entries meeting these requirements were determined by reference to the PDBTM register (Kozma *et al.*, 2013 ▸). Those structures within the globular set annotated as coiled coils by the SCOP classification (Andreeva *et al.*, 2020 ▸) were split into a coiled-coil set. For the resulting three data sets, the sequences of all structures were clustered using *CD-HIT* (Fu *et al.*, 2012 ▸), which also identified a representative sequence for each cluster using an identity cutoff of 20%. In order to remove redundancy among the structures in the three sets, only the structures of the representative sequences were kept in the data set. The *Phaser* expected log-likelihood gain (eLLG; Oeffner *et al.*, 2018 ▸) reflects the log-likelihood gain on intensity (LLGI) expected from a correctly placed model, and it can be used for the purpose of assessing the difficulty of solving a structure through MR using a given search model. In order to form a set of structures that are representative of the different ranges of difficulty that it is possible to encounter while solving different MR cases, the structures were organized into four bins according to their eLLG values obtained with a 40-residue polyalanine ideal helix as a search model and an expected r.m.s. value of 0.1. The ranges of these bins were set using the values indicated in the *Phaser* guidelines (Oeffner *et al.*, 2018 ▸): above 64, between 64 and 49, between 49 and 36, and between 36 and 25. This resulted in the final selection of 34 transmembrane (Supplementary Table S1), 31 globular (Supplementary Table S2) structures and 13 coiled coils. Owing to the small number of coiled-coil structures found in this way, we added increasingly difficult structures with eLLG values lower than 25 (a further eight) and included 25 structures that were used in previous studies (Thomas *et al.*, 2020 ▸). This resulted in the final set of 46 coiled coils used in this study (Supplementary Table S3), with structures outside the 2–3 Å resolution range. These include the structures with PDB codes 3hfe, 4dzk, 1g1j, 1y66, 3q8t and 2efr with resolutions better than 2 Å, the highest resolution being PDB entry 1y66 at 1.65 Å, and the structures with PDB codes 4u5t, 6gbr, 3mqb, 6bri and 4gkw with resolutions worse than 3 Å, the lowest resolution being PDB entries 4u5t and 4gkw at 3.30 Å. This final set of structures was distributed across the different eLLG bins as follows: 34% with an eLLG above 64, 15% with an eLLG between 64 and 49, 20% with an eLLG between 49 and 36, 15% with an eLLG between 36 and 25, and 16% with an eLLG below 25. The distribution by fold class (globular, transmembrane and coiled coil) is shown in Supplementary Table S4.

### 2.2. Creation of the helical ensembles   

In order to assess whether the structural divergence between the models that form an ensemble has effects on its effectiveness as a search model, both low-divergence (homogeneous) and high-divergence (heterogeneous) ensembles were created. This was performed by first performing a secondary-structure search of all of the structures in the PDB (Berman *et al.*, 2002 ▸) with *GESAMT* (Krissinel, 2012 ▸) using each of the eight ideal helices first used in *AMPLE* (Thomas *et al.*, 2015 ▸) as search queries. The top four hits within an r.m.s.d. of 0.5 Å across all C^α^ atoms of the helix in the case of the homogeneous ensembles and of between 0.5 and 1.0 Å for the heterogeneous ensembles were then structurally aligned with the original ideal helix to generate ensembles in the library (Supplementary Table S5). Finally, all side chains present in the models were removed in order to create polyalanine helices.

### 2.3. *B*-factor treatments of the helical ensembles   

**Figure 1:**
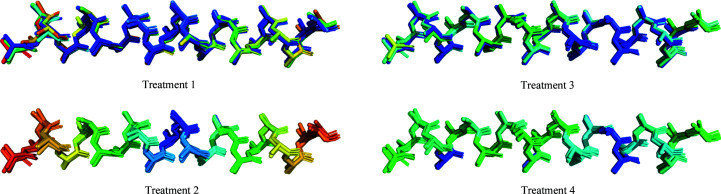
Depiction of the strategies used for the modification of *B* factors of the ensembles. Residue colours correlate with their assigned *B* factor, and follow a scale from blue for lower to red for higher going through green. All of the treatments are illustrated using the 25-residue homogeneous ensemble. Treatment 1: native *B* factors kept unmodified as in the native crystal structure. Treatment 2: a gradient of *B* factors is created through the helical ensemble. Treatment 3: each residue has a *B* factor proportional to its mean distance to the rest of the equivalent residues in the other four models. Treatment 4: each residue on the ensemble is set with a *B* factor proportional to the average distance of all of the models in that position.

*B* factors play an important role in the *Phaser* algorithm, as it bases the calculation of the structure factors on the normalized values of the *B* factors of the atoms present in the search model. This results in the downweighting of regions with higher *B* factors, in a similar manner to the way that ensemble-averaging calculations help *Phaser* to downweight the more variable regions of the ensemble (Read, 2001 ▸). In order to assess whether *B*-factor adjustments reflecting the variability across models in different regions of the ensemble could increase the effectiveness of the search model during MR searches, the residue *B* factors of the helical ensembles were modified using four different strategies (Fig. 1 ▸). Firstly, in *B*-factor treatment 1, the native *B* factors observed in the crystal structure were kept unmodified. In the case of *B*-factor treatment 2, the *B* factors were modified along a gradient, with values starting at 10.0 Å^2^ for the two residues located at the centre of the helix (central residue numbers were rounded up in the case of even helical sizes) and increasing to a limit of 90.0 Å^2^ towards the extremes. Uniform steps were used to increase the *B* factors between residues, and all of the atoms of each residue were assigned the same value. In treatments 3 and 4, *B* factors were modified according to the structural variance observed across the models of the ensemble at each residue position. To perform this, distances between all of the C^α^ atoms of the residues at equivalent positions across the models of the ensemble were measured. In the case of treatment 3, the mean distance between each member of the ensemble in turn and the rest of its equivalent residues was measured, and *B* factors were set for all atoms of each residue with a value of 80.0 Å^2^ if the distance was greater than 0.8 Å and a value of 10.0 Å^2^ if the average distance was less than 0.1 Å; a *B* factor resulting from the equation
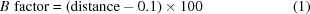
was used for any other value (see Supplementary Fig. S1 for a detailed view). Finally, in the case of treatment 4, the same *B* factor was set at each position across the residues of all five models of the ensemble. For each position in the ensemble, the average distance between all equivalent residues was measured and the *B* factor was set as stated for treatment 3.

### 2.4. Molecular replacement   

Each structure in the data set was trialled using two independent *AMPLE* MR runs, one using the original set of ideal helices (ideal helix mode) and the other using the new helical ensembles as an alternative (helical ensemble mode). *AMPLE* makes use of the *MrBUMP* pipeline (Keegan *et al.*, 2018 ▸), which in turn uses *Phaser* (McCoy *et al.*, 2007 ▸) for molecular replacement, *REFMAC*5 (Vagin *et al.*, 2004 ▸) for refinement and *SHELXE* (Thorn & Sheldrick, 2013 ▸) for density modification and C^α^ tracing. For the purpose of this study, the *Phaser* kill-time value was set to 24 h and in the case of coiled-coil folds translational noncrystallographic symmetry corrections in *Phaser* were turned off. *Phaser* calculated a variance-r.m.s. (VRMS) parameter for each of the input ensembles to optimize the calculation of its log-likelihood gain score (LLG) and improve the chance of picking out a correct solution (Randy Read, personal communication). This gave calculated VRMS values of between 0.2 and 1.5 depending on the ensemble, replacing the default value of 0.1 supplied by *AMPLE*.

To determine the success of the molecular-replacement attempts, initially it was determined whether the search model had been placed correctly by calculating the correlation coefficient between the electron-density map of the deposited experimental data and the map of the placed model (MapCC) using *phenix.get_cc_mtz_mtz* from the *Phenix* suite (Liebschner *et al.*, 2019 ▸). Search models were then considered to be correctly placed if this coefficient reached at least 0.2. Nevertheless, a correct placement does not always imply that it will be possible to trace the model and solve the unknown structure. In this particular aspect, the *SHELXE* correlation coefficient (CC) has been observed to be a reliable indicator of success for structures with a resolution of 2.5 Å or better (Thorn & Sheldrick, 2013 ▸). Thus, for those MR trials with a correctly placed search model, a *SHELXE* correlation coefficient (CC) of at least 25% was used as an additional criterion for success.

Owing to the special characteristics of coiled coils, the generally accepted metrics of success do not always correctly indicate an actual solution (Thomas *et al.*, 2015 ▸, 2020 ▸), so these cases required additional examination: to be judged successes, the following additional criteria, in addition to those above, had to be met. Firstly, it was determined whether each coiled-coil case was solved or not by following the same procedure as described in a previous *AMPLE* coiled-coil case study (Thomas *et al.*, 2020 ▸). Accordingly, for each structure, the solution with the highest ranking *SHELXE* correlation coefficient was used in automated model building with *Phenix AutoBuild* (Terwilliger *et al.*, 2008 ▸). This was performed using the *SHELXE* output build as the initial model, and it was tested both with and without the use of NCS for density modification. Successful solutions were then determined by an *R*_free_ value of below 0.45, the completeness of the model and a correlation coefficient between its 2*F*_o_ − *F*_c_ map and that of the deposited structure of above 0.60.

### 2.5. Computing resources and software versions   

Tests were carried out on a computing grid where each node was equipped with twin 8-core Intel Xeon E5-2660 SandyBridge processors running at 2.2 GHz and sharing 64 GB of memory. These processors were connected via QDR InfiniBand (40 GB s^−1^), with NFS storage running over this Infini­Band. Nodes were running the RedHat 6.2 Linux operating system.

All software used in the MR trials of this study corresponds to *CCP*4 version 7.073 (Winn *et al.*, 2011 ▸): *Phaser* version 2.8.2 (McCoy *et al.*, 2007 ▸), *REFMAC* version 5.8 (Vagin *et al.*, 2004 ▸) and *SHELXE* version 2019/1 (Usón & Sheldrick, 2018 ▸). The versions of *Phenix AutoBuild* and *phenix.get_cc_mtz_mtz* correspond to the *Phenix* suite version 1.17 (Liebschner *et al.*, 2019 ▸). Figures were created using *Matplotlib* version 2.2.5 (Hunter, 2007 ▸) and the *PyMOL* molecular-graphics system (version 2.4; Schrödinger).

## 3. Results and discussion   

### 3.1. Higher success rate with the ensemble library than the set of ideal helices   

To assess the performance of single model ideal helices against their ensemble counterparts, a set of 111 structures were selected as described above. To test performance across different types of structures, this set was composed of transmembrane, globular and coiled coils: 34, 31 and 46 structures, respectively. Using *AMPLE*’s ideal helix and helical ensemble modes, a solution was attempted for each of these structures using the original library of ideal helices and the members of the newly generated ensemble library as search models, respectively.

**Figure 2:**
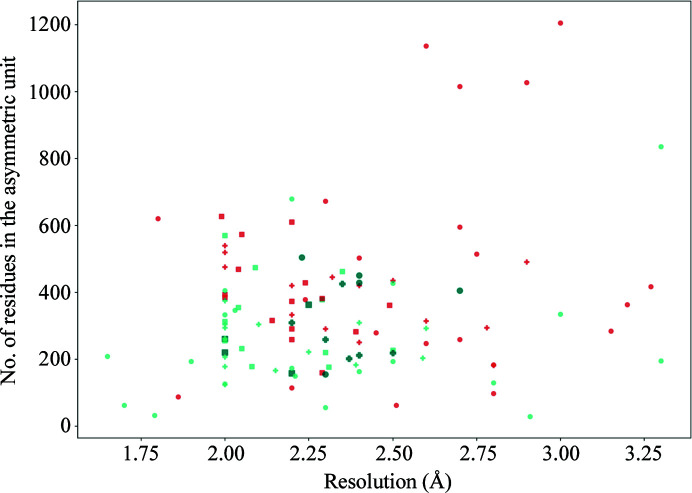
Distribution across different resolutions and numbers of residues in the asymmetric unit of the structures solved using ideal helices and ensembles for the three different data sets. Each point represents a case from the data set: transmembrane helical structures are represented with a cross, α-­globular structures with a square and coiled coils with circles. Orange points indicate cases that were not solved by either ideal helices or ensembles. Light turquoise points represent cases that were solved by both ensembles and single models. Darker turquoise points indicate cases that could only be solved by ensembles. The full results can be seen in Supplementary Table S6.

**Figure 3:**
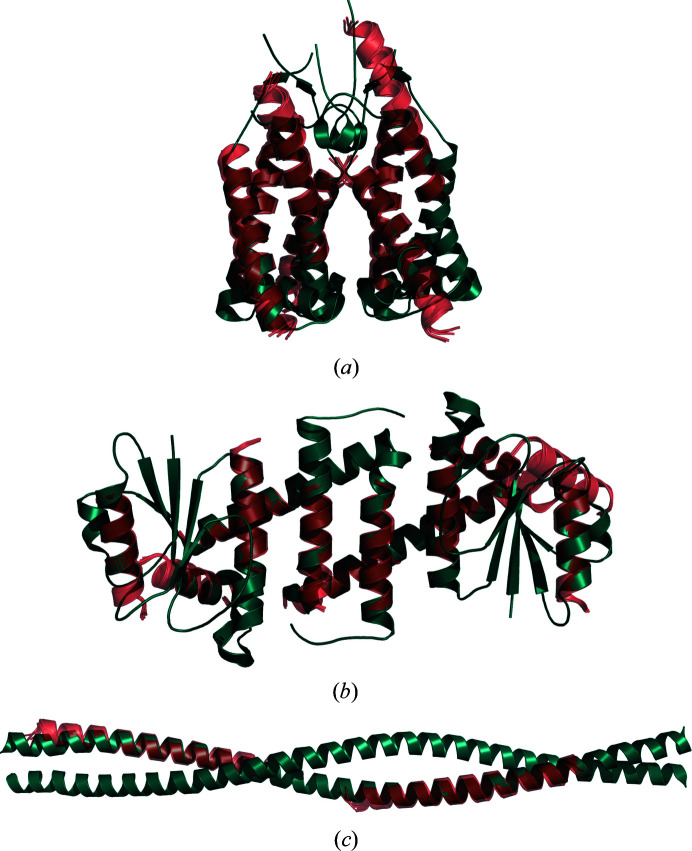
Examples of successful MR runs using members of the new library of helical ensembles. (*a*) illustrates the solution of the transmembrane structure PDB entry 4ri2, with seven placed copies of the 25-residue helical ensemble. (*b*) corresponds to the globular structure PDB entry 5mq8, with 12 placed copies of the 15-residue helical ensemble. (*c*) shows the solution for the coiled-coil structure PDB entry 1d7m, with two copies of the 35-residue ensemble. In all three figures the dark turquoise chains correspond to the deposited crystal structure and the orange chains to the MR-placed ensembles (only the first model of the ensemble is shown). Search models were made transparent to facilitate the visualization of the target structure.

For 46 of the 111 structures in the data set, at least one of the members of the original *AMPLE* ideal helix library was placed correctly by *Phaser*, and *SHELXE* was able to successfully build a model from this initial placement (Supplementary Table S6). The range of solutions achieved by these single model ideal helices is broad (Fig. 2 ▸), with solutions up to a resolution of 2.6 Å (PDB entry 6i6b), with up to 570 residues in the asymmetric unit (PDB entry 4fp4) and with an eLLG as low as 26 (PDB entry 5zle). The number of solutions achieved when using the new library of ensembles increases to 61, an increase of 30% on the total number of solutions achieved using ideal helices. Curiously, this increase was not uniform across the three fold types under study, with the highest increase being observed for transmembrane structures (40%), followed by globular (30%) and coiled-coil folds (25%). Additionally, the use of the new library of helical ensembles enabled *AMPLE* to solve increasingly challenging structures across the three folds, as in the case of PDB entry 1d7m, a coiled-coil structure with a resolution of 2.7 Å and 404 residues in the asymmetric unit, the globular structure with PDB code 5mq8, which has 325 residues in the asymmetric unit and a resolution of 2.25 Å, and the transmembrane structure with PDB code 4ri2, with a resolution of 2.35 Å and 412 residues in the asymmetric unit (Fig. 3 ▸). Encouragingly, this increase in the absolute number of solutions was achieved with no loss of prior solutions: all of the structures solved using single ideal helices were also solved with at least one member of the new ensemble library.

*SHELXE* CC can be used as an excellent guide to correct MR solutions in most cases with a resolution of better than 2.5 Å (Thomas *et al.*, 2015 ▸, 2020 ▸; Thorn & Sheldrick, 2013 ▸). Encouragingly, 90% of those search models that were placed correctly according to our primary success criterion, a MapCC of at least 0.2, reported a *SHELXE* CC higher than 25%. The high proportion of successful rebuilds achieved by *SHELXE* reflects the ability of its autotracing algorithm to correctly build the the main chain of a target with great accuracy. However, as is well known (Thomas *et al.*, 2015 ▸, 2020 ▸; Thorn & Sheldrick, 2013 ▸), this metric loses accuracy in cases with poor resolution or for coiled-coil folds. In recognition of this fact, Caballero *et al.* (2018 ▸) introduced an additional verification algorithm that validates the solution by the perturbation of the substructure that led to the solution and comparison of the figures of merit before and after this perturbation, but here, since the target structure is known, MapCC can be used to provide a definitive guide to correctness. In this regard, of the 71 structures that had *SHELXE* CC values above 25%, 12 were identified as MR failures when the MapCC was examined. The resolutions of these MR trials ranged between 1.67 and 3.2 Å, with an average of 2.2 Å. All corresponded to coiled-coil structures except for PDB entry 4rym, a transmembrane structure with a resolution of 2.8 Å.

**Figure 4:**
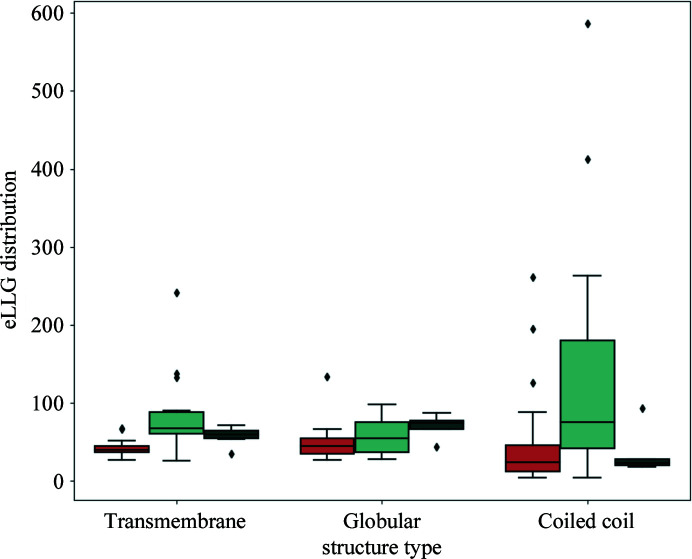
A box plot of the range of eLLGs for structures that could not be solved using either single model ideal helices or ensembles (orange), those that were solved using both single model ideal helices and ensembles (light turquoise), and those that could only be solved by *AMPLE* when using members of the new ensemble library as search models (dark turquoise). Box limits indicate upper and higher quartiles, whiskers indicate upper and lower bounds and the horizontal line in the middle of the box plot represents the median. Outliers are depicted as rhombi.

In order to quantify the extent to which using the new ensembles extends the range of *AMPLE* solutions towards increasingly difficult cases, the eLLG for each of the solved structures was calculated using a 40-residue ideal helix as a search model: this eLLG can be used as an indicator of the overall difficulty of a case, with lower values indicating greater difficulty. A comparison of the distribution of eLLG values between different categories of success was then made (Fig. 4 ▸). Although the numbers are relatively small, differing patterns in the eLLG values of the additional solutions achieved by the ensembles in each of the three fold classes were observed. Both transmembrane and coiled-coil structures that could only be solved by making use of the ensemble library have eLLGs within the bottom 25% of the base solutions obtained with ideal helices, an indication that using ensembles extends the range of solutions obtained using the *AMPLE* ideal helix mode towards increasingly difficult cases. Curiously, this pattern was not observed in the globular data set, where the structures that could only be solved using the members of the ensemble library present a high eLLG distribution, with most of them having eLLG values within the top 25% of the structures solved using the original set of ideal helices. Therefore, it appears that, in contrast to the other two fold types, rather than enabling *AMPLE* to solve increasingly difficult globular structures, using the new ensemble library yields solutions for those cases that could not be solved using ideal helices despite having relatively high eLLGs.

### 3.2. The optimal helical ensemble length varies with fold class   

The original ideal helix library first implemented in *AMPLE* had eight ideal helices, with a size range starting at five residues and extending to 40 residues with a five-residue step (Thomas *et al.*, 2017 ▸). In order to assess how the size of the search model affects the outcome of MR, the search models in the new ensemble library were grouped into size bins and the number of solved structures observed in each bin was measured across the three data sets (Table 1 ▸). Interestingly, the most successful search-model size was 25 residues for the transmembrane data set, 15 residues in the case of globular structures and 30 residues for coiled coils. These differences across the three data sets possibly reflect the different nature of these structures, as the thickness of most lipid bilayers can accommodate helices of approximately 20–30 residues (Hildebrand *et al.*, 2004 ▸), helical regions of globular proteins have a broad range of sizes and coiled coils tend to consist of very elongated helices. It is also possible to observe that search models with only ten residues or fewer were consistently the least successful across the three data sets, something that is especially noticeable in the case of transmembrane structures, where no solutions were observed when using five-residue helices.

### 3.3. Ensemble heterogeneity does not affect search-model effectiveness   

For each possible combination of helix size and *B*-factor treatment, both a low-divergence homogeneous ensemble and a more heterogeneous ensemble with high variability between its models were created. In order to assess whether the structural similarity between the different models that comprise the ensemble has an effect on the effectiveness of the search model, results with these two versions of ensembles were analysed (Table 2 ▸). This revealed no significant differences in the number of solutions obtained using homogeneous ensembles and their heterogeneous counterparts in any of the three structural folds, an indication that search-model effectiveness is not affected by changes in the level of ensemble heterogeneity within the range of values being tested in this study.

Despite not having observed significant effects of the ensemble heterogeneity on the total number of solved structures, a comparison of the effectiveness of homogeneous and heterogeneous search models revealed that the success ratio of these two types of ensembles varies across different resolution ranges (Supplementary Fig. S2). Interestingly, homogeneous ensembles appeared to be more successful for structures with resolutions both worse than 2.75 Å and better than 2.00 Å, while it was not possible to appreciate any tendency for the rest of the structures within the 2.00–2.75 Å range.

### 3.4. A minimal set of ensembles can be used without any solution loss   

**Figure 5:**
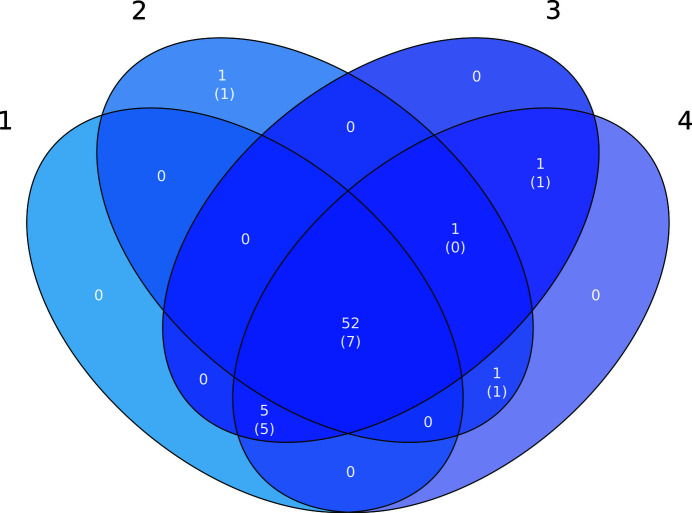
Venn diagram showing the number of solved structures achieved with each *B*-­factor treatment. The black numbers indicate the *B*-factor treatments (see the text for details). The total numbers of solutions are indicated with white numbers, and the numbers of solutions that could only be achieved using members of the ensemble library and not the original *AMPLE* ideal helices are given in parentheses.

In order to assess whether there was any level of redundancy among the different ensemble *B*-factor treatments in the new library, the structures solved by each ensemble were gathered and the common solutions across different ensemble preparations were compared (Fig. 5 ▸). It is interesting to observe that the four *B*-factor treatments shared 52 of the 61 solutions achieved by the ensemble library, indicating a high level of similarity in the performance of these ensemble treatments. Interestingly, this similarity was also observed across different resolution ranges, as no evident differences in performance were revealed after performing an analysis of the search-model effectiveness across different resolutions (Supplementary Fig. S3). Nevertheless, not all ensembles contributed in the same way to solving the additional targets that could not be solved using the original *AMPLE* library of single model ideal helices. Thus, only five of the 17 newly solved targets succeeded with ensembles from all four *B*-factor treatments, indicating the importance of screening the broad spectrum of ensemble treatments to gain elusive solutions that could not be solved with the original ideal helices. However, it is important to note that a reduced set of ensembles could still be created if required without losing any of those additional solutions, as search models with *B*-factor treatments 2 and 3 cover the full range of extra solutions obtained using ensembles.

### 3.5. A minimal set of ensembles yields solutions faster than the original library of ideal helices   

The new library of ensembles consists of 64 members, which represents an eightfold increase in the number of search models over the original library of ideal helices. In order to assess whether this increase in library size translates into an increase in the computing time consumed by *AMPLE*, we compared the timings of both approaches in those cases where at least one of the members of both libraries yielded a solution. To do this, we registered the time required to reach a solution by using previously recorded timings of MR runs and simulating four different MR strategies. The first simulation (simulation 1) corresponded to the time spent by *AMPLE* using the new library of helical ensembles, while the second simulation corresponded to the use of the original set of ideal helices (simulation 2). In these first two simulations, the search models were ordered by increasing chain size and no specific priority was set among the different *B*-factor treatments in the new library. Additionally, we were also interested in extending this comparison to a minimal subset of ensembles, which was created using the observations made about the performance of the different ensembles in the previous section. Since ensemble heterogeneity had no major effects on search-model success, only the homogeneous ensembles were taken into the minimal subset (Table 2 ▸). Additionally, since five- and ten-residue ensembles were observed to be the least successful (Table 1 ▸), helical ensembles of this size were removed from the third simulation. Regarding *B*-factor treatments, the results showed that there is no solution loss when only using ensembles with *B*-factor treatments 2 and 3 (Fig. 5 ▸), which were taken into the minimal subset of ensembles. All of these changes resulted in the creation of a subset of 12 ensembles, which were trialled in the third and fourth simulations. These two last simulations differed in the order in which the ensembles were trialled. In the third simulation, the search-model sorting was determined by the overall success rates previously observed for each ensemble size, independently of the fold class, and the search models with the most successful sizes were trialled first (simulation 3). Finally, since we observed that the optimal ensemble size varies across fold types (Table 1 ▸), we implemented a fold-specific ordering in the fourth simulation (simulation 4).

**Figure 6:**
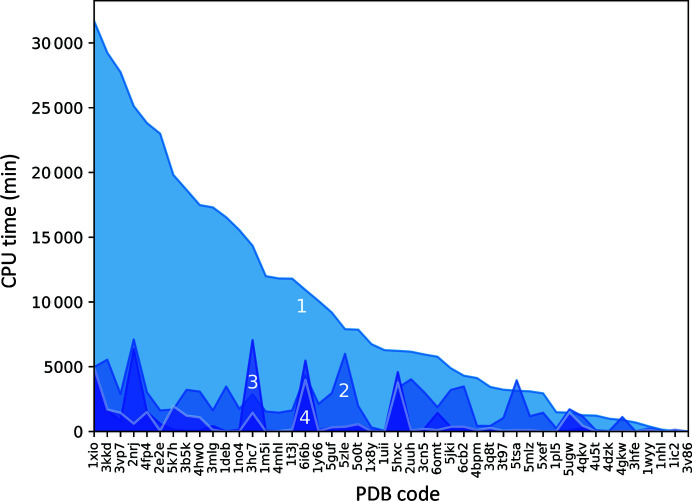
Elapsed time obtained by simulations based on job timings before *AMPLE* reaches the first solution using as search models the members of the new ensemble library (1), the original library of ideal helices (2) and a minimal subset of the new ensemble library using fold-independent search-model sorting (3) and fold-specific ordering (4).

Interestingly, the increase in the number of search models in the new ensemble library translates into an increase in the time necessary before a solution is found in simulation 1 when compared with the time required by *AMPLE* when the original ideal helices are used in simulation 2 (Fig. 6 ▸). Nevertheless, when using the minimal subset of ensembles, simulations 3 and 4 show that *AMPLE* was not only able to match the time performance of the single-model library, but was also able to yield a solution more rapidly across most targets in the data set. This reveals a further advantage of using the new ensemble library: it not only achieves more solutions than the original ideal helix library, but also reaches a solution more rapidly in those cases where the solution can be found by the original library. Interestingly, despite having observed that ensemble sizes have different success rates depending on the fold class of the unknown structure, we observed only minor improvements in the time elapsed before a solution is found when a fold-specific search-model ordering is used, as the differences between simulations 3 and 4 are minimal in most cases.

## 4. Conclusion   

Here, we have presented a new take on the concept of using helices as search models by making use of a new set of 64 helical ensembles. The use of the new library of ensembles resulted in a 30% increase in the total number of solutions compared with a library of ideal helices, an increase that was variable across the three folds under study. Having observed no solution loss when using ensembles, we strongly encourage the use of this new library of search models as an alternative to ideal helices. These findings agree with observations made in previous studies, where clustering several search models into ensembles outperformed the individual use of these models (Keegan *et al.*, 2018 ▸; Rigden *et al.*, 2002 ▸; Simpkin *et al.*, 2020 ▸) as their structural variability can be used to statistically weight sets of structure factors (Read, 2001 ▸). We also observed that some solutions required the use of ensembles modified with a series of *B*-factor treatments reflecting the structural variability across the models of the ensemble. These findings stress the importance of *B* factors in the *Phaser* algorithm, as the structure factors are computed based on the normalized *B* factors of the atoms in the supplied search model before actual MR operations begin.

Based on observations made about the efficacy of our new search models, we have been able to create a minimal subset of 12 ensembles without any solution loss compared with the full-size library, revealing that our new approach does not require the intensive use of computational resources to improve upon results obtained with previous methods. Thus, we believe that the use of our minimal subset of helical ensembles can be an alternative MR route to the more time-consuming approaches that are required when no homologous structures can be found, and therefore we have made this subset available to *AMPLE* users by activating the -helical_ensembles keyword. Alternatively, *AMPLE* users can still use the full set of 64 ensembles, if desired, by setting the keyword -helical_ensembles_set to full. In contrast to some of these approaches that rely on precise tertiary-structure predictions to produce suitable search models (Keegan *et al.*, 2015 ▸; Rigden *et al.*, 2018 ▸; Simkovic *et al.*, 2016 ▸), our take on fragment-based MR only requires the presence of α-helices within the unknown structure, which are known to be the most reliably predicted regular secondary-structure elements (Cuff & Barton, 2000 ▸). We expect that our new set of ensembles will also be able to provide assistance with the other commonly observed limitations of fragment-based MR, especially with regard to the completion of partial MR solutions, that are commonly observed in this approach (Millán *et al.*, 2020 ▸).

## Supplementary Material

Supplementary Tables. DOI: 10.1107/S205979832001133X/rr5198sup1.xlsx

Supplementary Figures. DOI: 10.1107/S205979832001133X/rr5198sup2.pdf

## Figures and Tables

**Table 1:** Analysis of the percentage of solved structures per ensemble size across the three fold types Each entry represents the percentage of solutions that can be obtained using exclusively ensembles of each specific size.

	Fold type		
Search-model size	Transmembrane	Globular	Coiled coils
5	2	0	4
10	9	3	9
15	18	15	12
20	17	17	13
25	15	21	15
30	14	17	16
35	14	14	15
40	10	13	15

**Table 2:** Analysis of the percentage of solved structures per ensemble divergence across the three fold types Each entry represents the percentage of solutions that can be obtained using exclusively ensembles of each specific divergence.

	Fold type		
Ensemble divergence	Transmembrane	Globular	Coiled coils
Heterogeneous	50	48	52
Homogeneous	50	52	48
