# Supplementary material for: Helical ensembles outperform ideal helices in molecular replacement
Source: Acta Crystallogr D Struct Biol. 2020 Sep 16;76(Pt 10):962–70. doi: 10.1107/S205979832001133X (PMC7543657; doi:10.1107/S205979832001133X)
Supplement: Supplementary file 2 [file d-76-00962-sup2.pdf]

STRUCTURAL
BIOLOGY

Volume 76 (2020)

Supporting information for article:

Helical ensembles outperform ideal helices in molecular replacement

Filomeno Sánchez Rodríguez, Adam J. Simpkin, Owen R. Davies, Ronan M. Keegan and Daniel J. Rigden

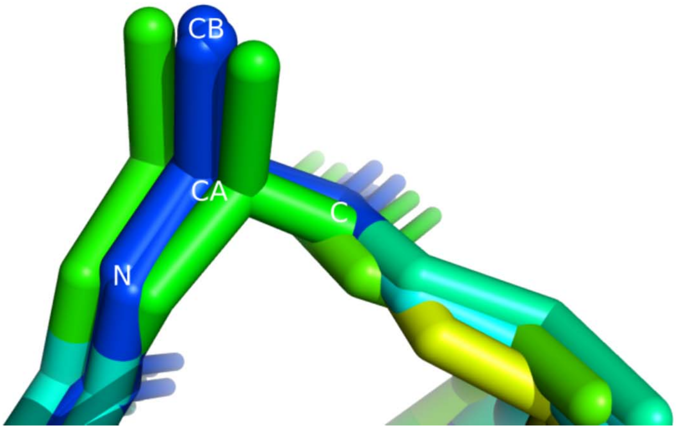

Figure S1 Detailed view of the 8th alanine residue of the 25 residue long homogenous ensemble where the B-factor has been modified as in treatment 3: the B-factor value is proportional to the average distance between each residue and the other residues at the equivalent positions of the other four models. Thus it is higher - green rather than blue - for outlying residues at this position in the ensemble.

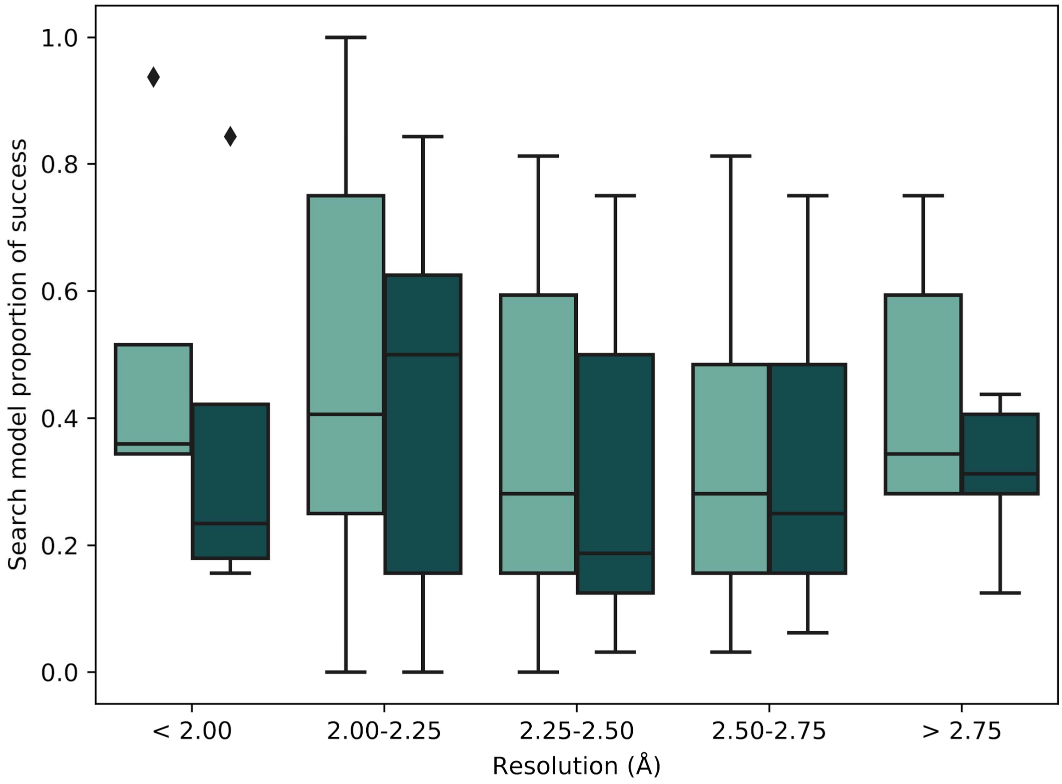

Figure S2 Distribution of the proportion of search model successes across different resolution ranges between homogeneous ensembles (light turquoise boxes) and heterogeneous ensembles (dark turquoise boxes). The proportion of search model successes was defined as the fraction of search model ensembles that succeeded in solving the test structure. Box limits indicate upper and higher quartiles, whiskers indicate upper and lower bounds and a horizontal line in the middle of the box plot represents the median. Outliers are depicted as a rhombi.

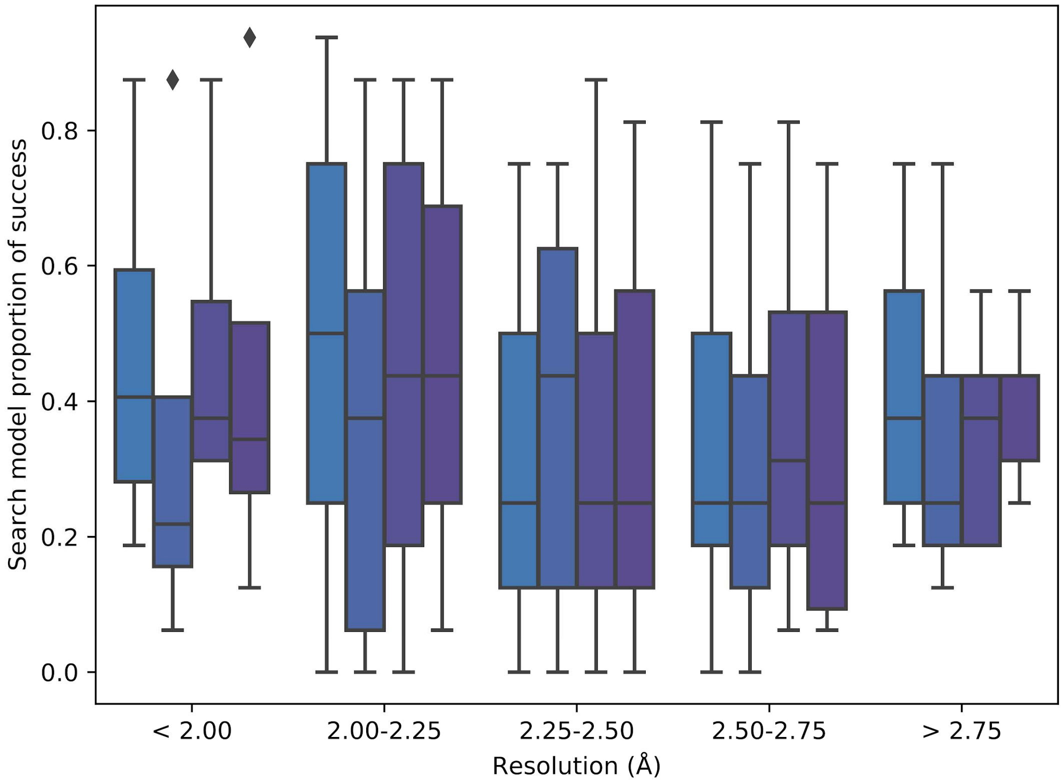

Figure S3 Distribution of the proportion of search model successes across different resolution ranges between different B-factor treatments -from blue to purple-: treatment 1, 2, 3 and 4. The proportion of search model successes was defined as the fraction of search model ensembles that succeeded in solving the test structure. Box limits indicate upper and higher quartiles, whiskers indicate upper and lower bounds and a horizontal line in the middle of the box plot represents the median. Outliers are depicted as a rhombi.
